# Study on Laser-Electrochemical Hybrid Polishing of Selective Laser Melted 316L Stainless Steel

**DOI:** 10.3390/mi15030374

**Published:** 2024-03-11

**Authors:** Jun Liu, Chunbo Li, Huan Yang, Jiani Liu, Jiayan Wang, Leimin Deng, Licun Fang, Can Yang

**Affiliations:** 1 Sino-German College of Intelligent Manufacturing, Shenzhen Technology University, Shenzhen 518118, China; 20461439087@stu.wuz.edu.cn (J.L.); lichunbo@sztu.edu.cn (C.L.); yanghuan@sztu.edu.cn (H.Y.); 15942537602@163.com (J.L.); jiayan_w1210@163.com (J.W.); 2 Wuhan National Research Center for Optoelectronics, Huazhong University of Science and Technology, Wuhan 430074, China; dlm@hust.edu.cn

**Keywords:** selective laser melting, 316L stainless steel, laser-electrochemical, surface roughness

## Abstract

The process of forming metal components through selective laser melting (SLM) results in inherent spherical effects, powder adhesion, and step effects, which collectively lead to surface roughness in stainless steel, limiting its potential for high-end applications. This study utilizes a laser-electrochemical hybrid process to polish SLM-formed 316L stainless steel (SS) and examines the influence of process parameters such as laser power and scanning speed on surface roughness and micro-morphology. A comparative analysis of the surface roughness, microstructure, and wear resistance of SLM-formed 316L SS polished using laser, electrochemical, and laser-electrochemical hybrid processes is presented. The findings demonstrate that, compared to laser and electrochemical polishing alone, the laser-electrochemical hybrid polishing exhibits the most significant improvement in surface roughness and the highest material wear resistance. Additionally, the hybrid process results in a surface free of cracks and only a small number of tiny corrosion holes, making it more suitable for polishing the surface of 316L SS parts manufactured via SLM.

## 1. Introduction

Additive manufacturing (AM), also known as 3D printing, is an advanced technology for directly fabricating digital models into solid parts by accumulating materials layer by layer. It has garnered increasing attention due to its advantages in manufacturing high-strength and complex parts [1,2,3]. Among the various technologies in AM, SLM stands out for its ability to prepare high-strength, fine, and complex structures by melting metal powders in a predetermined scanning path with a high-energy laser beam, followed by rapid solidification to obtain the desired solid part [4,5].

The 316L austenitic stainless steel is widely utilized in the fields of chemistry, marine engineering, food, and biomedicine due to its excellent mechanical properties and corrosion resistance [6,7,8,9]. Compared to conventional processes, SLM offers numerous advantages in preparing 316L SS, including high material utilization and the ability to fabricate complex structures within short fabrication cycles [10,11,12]. However, the inherent spheroidization effect, powder adhesion, and step effect in SLM result in poor surface roughness of the prepared parts [13,14,15,16], necessitating post-treatment and polishing to meet application requirements.

Currently, the processes employed for surface polishing of SLM additively manufactured 316L SS components primarily consist of mechanical, chemical, laser, and electrolytic polishing [17,18,19,20]. Low efficiency, difficulty in processing complex internal surfaces, and environmental pollution hinder applications of mechanical and chemical polishing. Laser polishing offers numerous advantages, such as being non-contact, non-polluting, and highly efficient [21,22,23]. However, the thermal effect generated during laser polishing is prone to causing recast layers and microcracks [24,25]. Electrochemical polishing has been widely utilized for the fine treatment of metal surfaces and can achieve a mirror finish for initial surfaces with a roughness (Ra) of about 1 µm [26,27]. However, due to the substantial surface roughness of 316L SS components prepared using SLM (Ra > 5 µm), conventional electrochemical polishing leads to non-selective and non-uniform smoothing/removal of the surface structure [28] and can even damage its original structure.

In order to address the challenges posed by individual manufacturing processes in terms of processing efficiency and quality, hybrid machining technology is increasingly being applied in high-end manufacturing fields [29,30]. Among these, laser and electrochemical hybrid machining technology integrates the advantages of high laser machining efficiency, flexibility, and the superior surface quality of electrolytic machining, making it a precision machining technology of great interest both domestically and internationally. Wang et al. [31] proposed the use of a laser–electrochemical hybrid machining process for small-hole machining. The study’s results demonstrated that laser assistance can increase the current density of the electrochemical machining area, thereby improving the electrochemical material removal rate. The machining accuracy and material removal rate were increased by 60.7% and 122.7%, respectively. Lescuras et al. [32] demonstrated that pulsed lasers can enhance the accuracy of machined edges. Silva et al. [33] developed a mathematical model to account for the localization effect of lasers in the electrochemical dissolution process. The results indicated that laser-assisted machining increased the removal rate by 54% and accuracy by 38% compared to electrochemical machining alone. Additionally, Silva et al. [34] showed that microcavities can be machined with high efficiency and without thermal damage using the laser–electrochemical hybrid addition technique.

There have been studies on the processing of metal micropores and microcavities using the laser–electrochemical hybrid process, but the polishing of large-area metals has not been addressed. Therefore, to address the current challenges associated with single-polishing technology in terms of processing efficiency and surface quality, this study proposes using the laser-electrochemical hybrid process to polish SLM-formed metal components. The study aims to investigate the effects of different process parameters on surface roughness and micro-morphology. Additionally, this study compares the surface roughness, microstructure, and mechanical properties of SLM-formed 316L SS after polishing using laser, electrochemical, and laser–electrochemical hybrid processes.

## 2. Experimental Method

### 2.1. Experimental Materials

**Figure 1:**
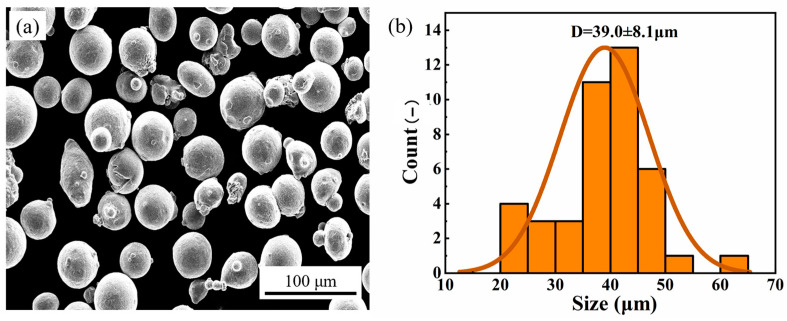
The 316L SS powder: (**a**) SEM morphology, (**b**) particle size distribution.

The experimental material utilized for SLM additive manufacturing, 316L SS powder, was provided by Hart 3D. Table 1 presents the main chemical composition of the powder. The powder morphology, depicted in Figure 1a, shows that the 316L SS powder is spherical, which facilitates part formation. The particle size of the powder ranges from 15 to 65 μm, with an average diameter of 39.0 μm and a standard deviation of 8.1 μm (Figure 1b).

A 15 × 15 × 3 mm³ 316L SS batch was prepared under a nitrogen environment using SLM equipment (Daqo Laser Co., Ltd., SLM-100, Shenzhen, China). The laser power was 180 W, the layer thickness was 0.03 mm, the scanning speed was 300 mm/s, and the laser scanning direction of the adjacent formed layers differed by 67° during the SLM process. Following preparation, the samples were cut from the substrate with an EDM cutter and subsequently cleaned sequentially in ethanol and deionized water using ultrasonication for 10 min to remove residual powder particles.

### 2.2. Experimental Device

**Figure 2:**
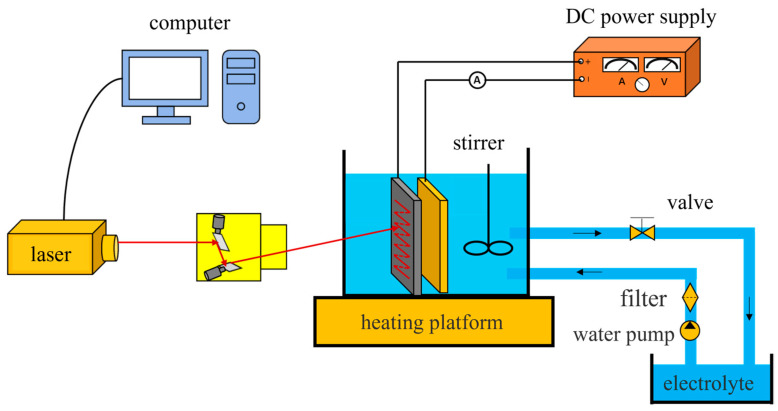
Laser–electrochemical hybrid polishing experimental system.

The laser-electrochemical hybrid polishing experimental system, as depicted in Figure 2, primarily consists of a laser processing system and an electrochemical processing system. The laser processing system comprises a laser, scanning microscope, and industrial control machine. The electrochemical processing system includes a DC power supply, electrolytic tank, electrode fixture, cathode, metal anode workpiece, and electrolyte. The anode workpiece material is SLM-formed 316L SS, with dimensions of 15 × 15 × 3 mm^3^. The cathode is a lead plate, and the electrolyte is a mixture of phosphoric acid, sulfuric acid, and deionized water in a volume fraction ratio of 6:3:1, kept at a temperature of 60 °C, with a processing area of 5 × 5 mm^2^.

### 2.3. Microstructure and Mechanical Property Test Methods

The surface roughness of the samples was assessed using a 3D laser confocal microscope (Olympus, OLS5000, Tokyo, Japan), while the surface morphology was examined with a scanning electron microscope (Carl Zeiss, GeminiSEM300, Oberkochen, Germany).

Surface hardness was characterized using an indentation Vickers hardness tester. Each surface underwent five measurements, and the average value was taken as the specimen’s average microhardness, employing a pressurized load of 300 g and a holding time of 15 s.

The wear resistance of the samples was evaluated using a reciprocating friction and wear tester (Yihua, MXW-1, Jinan, China). A GCr15 steel ball with a diameter of 5 mm was employed as the friction vice, with experimental parameters set as follows: experimental load of 10 N, reciprocating distance of 10 mm, frequency of 2 Hz, and test duration of 20 min.

## 3. Results and Discussion

### 3.1. Effect of Laser Power Parameters on Surface Roughness

**Figure 3:**
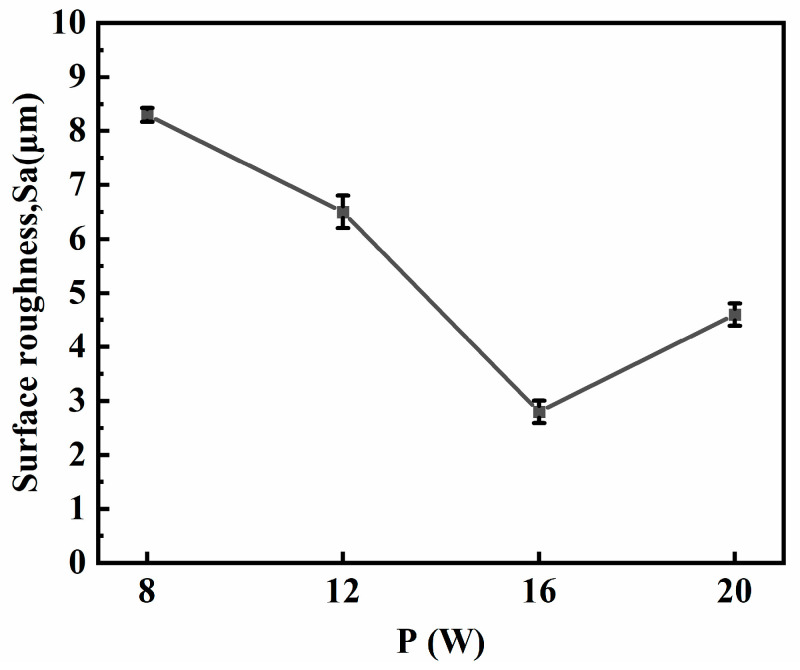
Surface roughness changes with laser power.

Figure 3 illustrates the impact of laser power on material surface roughness. The experiment employed laser powers of 8 W, 12 W, 16 W, and 20 W, with a DC power supply current of 4 A and a processing time of 3 min. The results indicate that as laser power increases, surface roughness initially decreases, followed by an increase. At 16 W, the roughness reaches a minimum of 2.8 μm.

**Figure 4:**
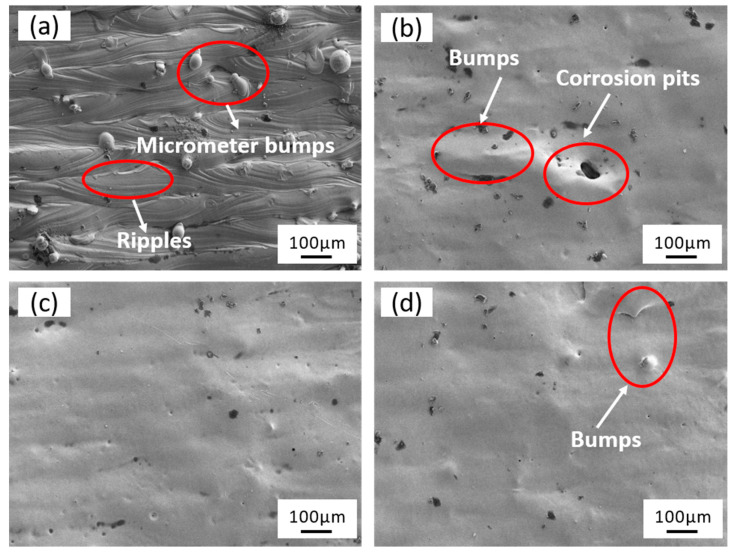
Surface microstructure: (**a**) the original samples (**b**), (**c**), and (**d**) laser power is 12 W, 16 W, and 20 W, respectively.

The microscopic surface morphology of the laser–electrochemical hybrid polished specimen under different powers is depicted in Figure 4. The original surface of SLM-formed 316L SS exhibits roughness with micron-sized bumps and ripples (Figure 4a). At 12 W, the surface displays bumps and corrosion pits due to the low laser power and material removal rate, making it unable to eliminate micron-level surface bumps (Figure 4b) completely. When the laser power is set to 16 W, the micron-level bumps are entirely eliminated, rendering the rough surface smooth (Figure 4c). However, at 20 W, the increased formation of bubbles at the solid–liquid interface leads to uneven polishing and the re-emergence of micron-sized bumps, resulting in increased roughness (Figure 4d).

### 3.2. Effect of Laser Scanning Speed Parameters on Surface Roughness 

**Figure 5:**
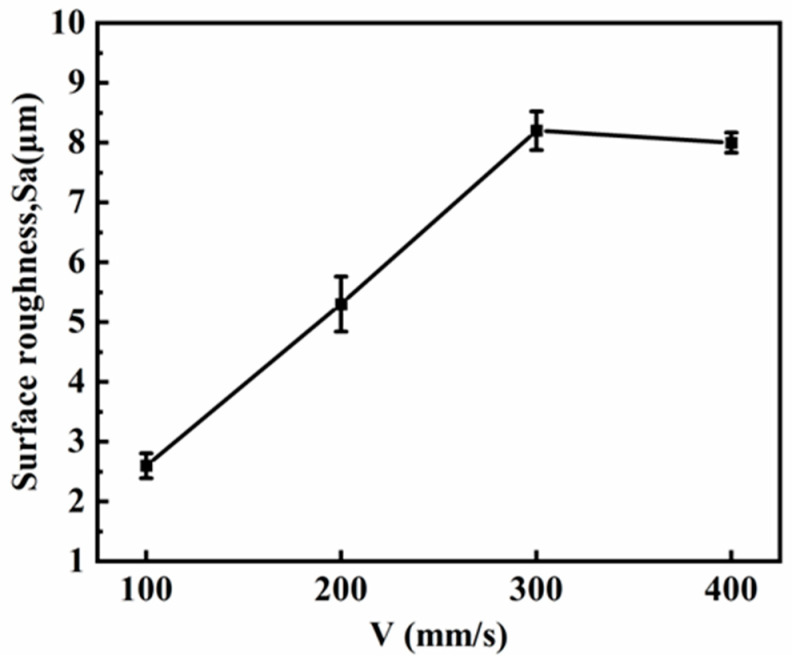
Surface roughness changes with scanning speed.

Figure 5 depicts the impact of laser scanning speed on surface roughness. The experiment encompassed a scanning speed range of 100 mm/s to 400 mm/s, with a DC power supply current of 5 A and a processing time of 3 min. The results indicate that specimen surface roughness gradually rises as scanning speed increases. However, within the scanning speed range of 300–400 mm/s, surface roughness exhibits no significant change.

**Figure 6:**
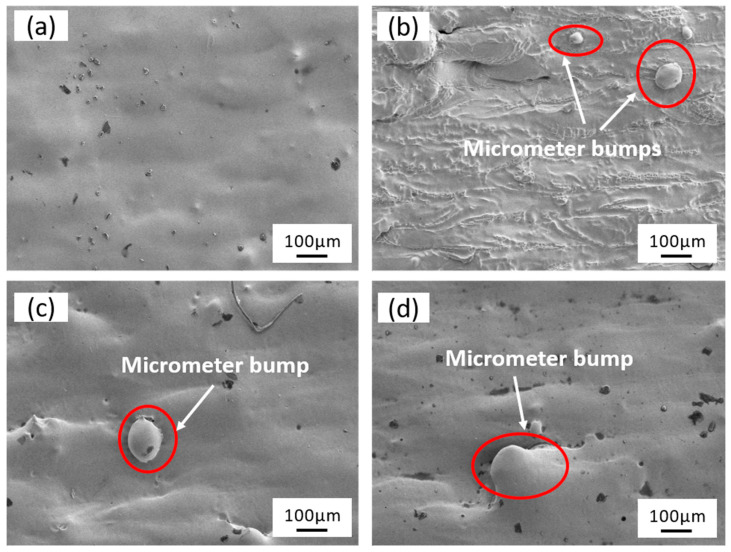
Surface microstructure: (**a**), (**b**), (**c**), and (**d**) scanning speeds were 100 mm/s, 200 mm/s, 300 mm/s, and 400 mm/s, respectively.

As illustrated in Figure 6, the surface micro-morphology of the specimens after laser-electrochemical hybrid polishing at different scanning speeds is presented. At a scanning speed of 100 mm/s (Figure 6a), compared to the initial sample (Figure 4a), the disappearance of surface ripples and micrometer-sized bumps is evident, resulting in a relatively flat state. With increasing scanning speed, surface undulations become more pronounced, giving rise to a rough appearance, while micrometer-sized bumps remain observable (Figure 6b–d).

### 3.3. The Effect of Laser Processing Time on Surface Roughness

**Figure 7:**
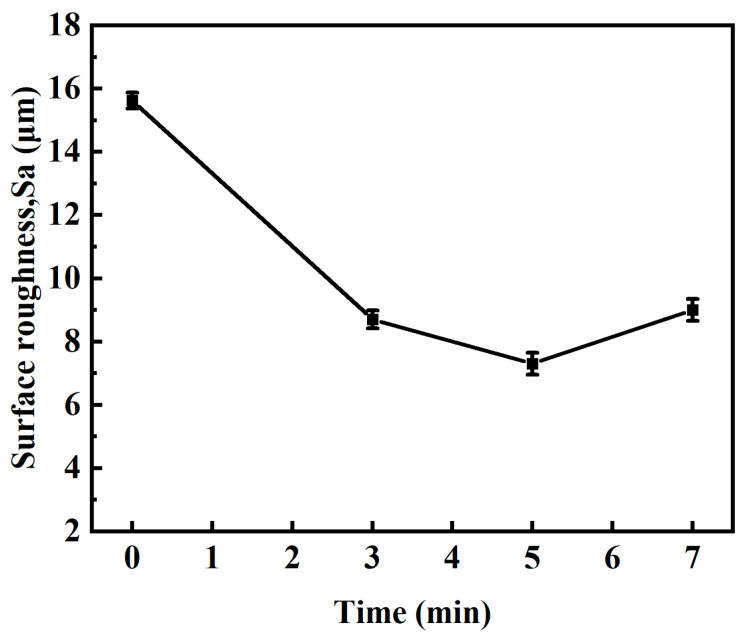
Variation of surface roughness with machining time.

Figure 7 presents the surface roughness evolution of the laser-electrochemical hybrid polished specimen over time. The time range utilized spans 3–7 min, with a current of 5 A, laser power of 16 W, scanning speed of 100 mm/s, and repetition frequency of 40 kHz. Experimental findings reveal that surface roughness decreases before ascending as time progresses, reaching its lowest point at 5 min of processing time, achieving a value of 1.9 μm.

**Figure 8:**
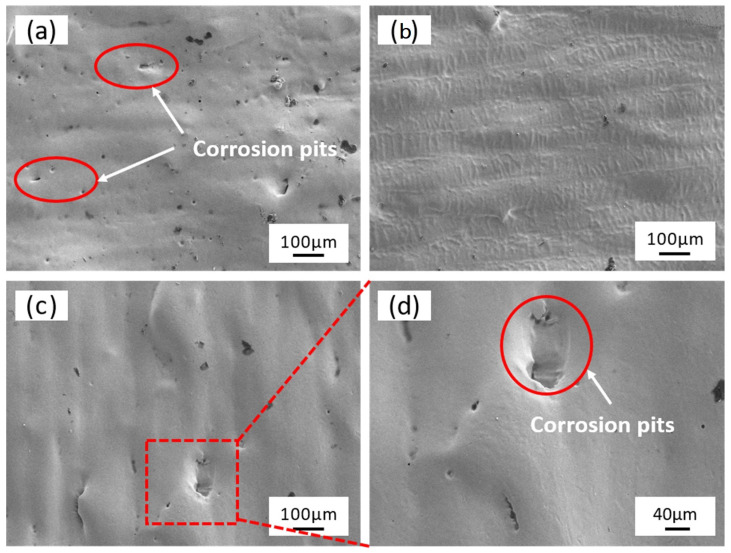
Surface microstructure: (**a**), (**b**), and (**c**) the processing time is 3 min, 5 min, and 7 min, respectively, and (**d**) is the enlarged picture of (**c**).

Figure 8 shows the microscopic surface morphology of the laser-electrochemical hybrid polished specimen at various time intervals. At 3 min of processing time, compared to the initial sample (Figure 4a), the surface ripples and micrometer-sized bumps are entirely eradicated (Figure 8a), albeit with a noticeable presence of corrosion pits. Conversely, at 5 min of processing time (Figure 8b), material surface flatness is further enhanced, with clearly visible laser processing traces and a diminished presence of corrosion pits. Upon further extension of the polishing time to 7 min, overcorrosion of the material surface becomes apparent, accompanied by larger corrosion pits and the resurgence of micrometer-sized bumps, resulting in increased roughness (Figure 8c,d).

### 3.4. Comparison of the Effect of Laser/Electrochemical and Laser-Electrochemical Hybrid Polishing Processes

**Figure 9:**
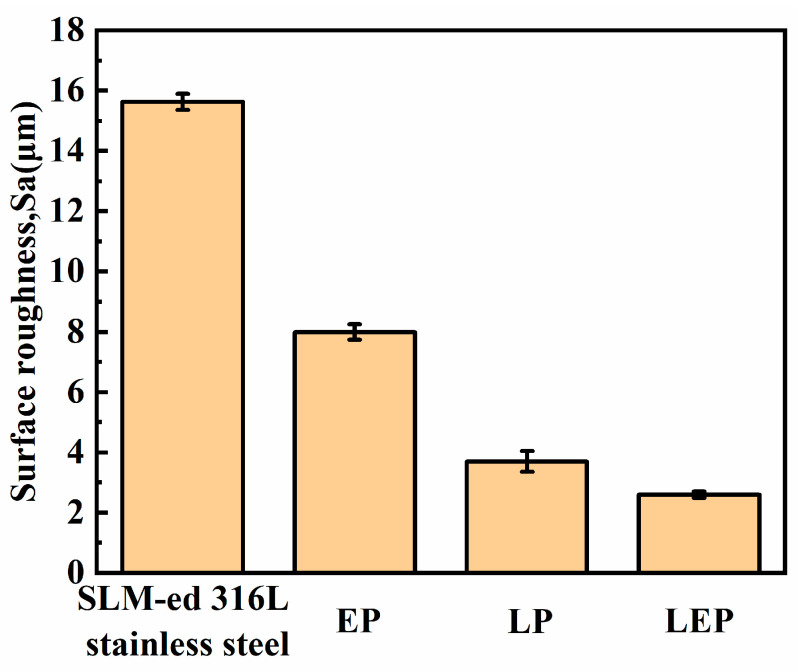
Surface roughness of SLM-formed 316L SS before and after polishing.

Figure 9 exhibits the impact of laser polishing (LP), electrochemical polishing (EP), and laser-electrochemical hybrid polishing (LEP) on SLM-formed 316L SS, focusing on changes in surface roughness. To ensure rapid and efficient polishing, all processes were set to a duration of 3 min. Table 2 presents the processing parameters. Figure 9 illustrates that electrochemical, laser, and laser–electrochemical hybrid polishing processes result in reduced surface roughness, decreasing from an initial 15.63 μm to 8.2 μm, 3.7 μm, and 2.6 μm, respectively, representing reductions of 47.5%, 76.3%, and 83.4%. It is evident that laser–electrochemical hybrid polishing yields the best results, followed by laser polishing, with electrochemical polishing exhibiting the least-favorable outcomes.

**Figure 10:**
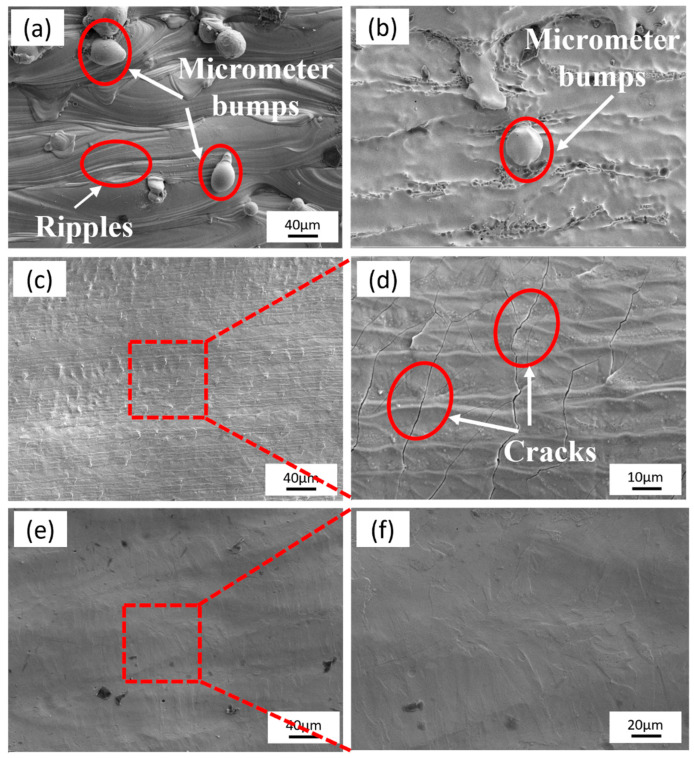
Surface microstructure before and after polishing: (**a**) original sample, (**b**) electrolytic-polishing sample, (**c**,**d**) laser-polishing sample, (**e**,**f**) laser–electrochemical hybrid polishing sample.

Figure 10 shows the microscopic surface morphology of the specimens after undergoing the three polishing processes. Figure 10a presents the original rough surface of SLM-formed 316L SS samples. Following electrochemical polishing, micrometer structures with lower roughness were removed, but larger micrometer-sized bumps persisted, accompanied by the emergence of corrosion pits in certain areas (Figure 10b). Laser polishing resulted in a substantial improvement in surface roughness; however, thermal stresses during the process led to the formation of microcracks (Figure 10c,d). The surface of the laser-electrochemical hybrid polished samples appeared flatter compared to the two individual polishing processes. The cooling effect of the polishing solution prevented re-melting and microcracking, significantly reducing the number of corrosion pits (Figure 10e,f).

### 3.5. Comparison of Microhardness of Laser/Electrochemical and Laser-Electrochemical Hybrid Polishing

**Figure 11:**
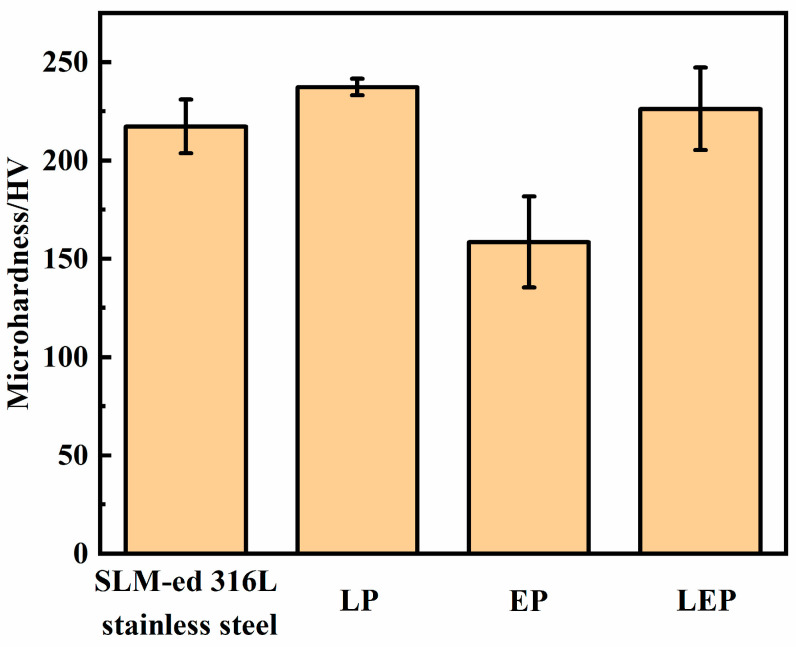
Sample microhardness distribution before and after polishing.

**Figure A2:**
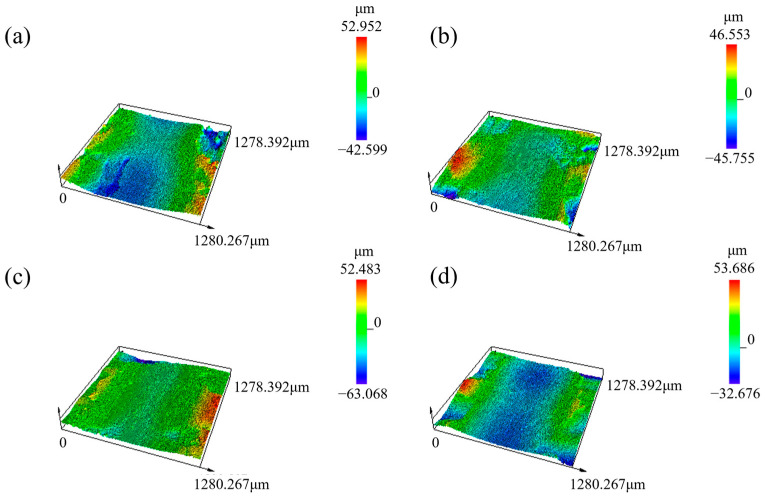
3D image of sample wear surface: (**a**) SLM-ed 316L stainless steel sample, (**b**) EP sample, (**c**) LP sample, and (**d**) LEP sample.

Figure 11 presents the microhardness of the sample surface following polishing via different processes. Microhardness test pictures are shown in Figure A2 in Appendix A. The average hardness of the original surface of SLM-formed 316L SS samples measured 217.3 HV. Subsequent to polishing using laser, electrochemical, and laser-electrochemical hybrid processes, the microhardness of the 316L SS surface was recorded at 237.3 HV, 158.6 HV, and 226.2 HV, respectively. The microhardness of the samples subjected to laser and laser-electrochemical hybrid polishing exhibited an increase, whereas the microhardness of the specimen surface following electrochemical polishing decreased. This phenomenon may be attributed to the higher hardness of the top of the samples compared to the bottom. Electrochemical polishing dissolved the top of the samples with high hardness, resulting in lower overall hardness.

### 3.6. Comparison of Wear Resistance of Laser/Electrochemical and Laser-Electrochemical Hybrid Polishing

#### 3.6.1. Friction Coefficient

**Figure 12:**
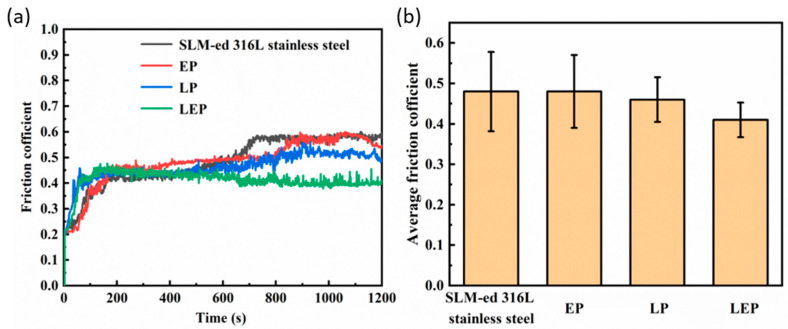
Samples before and after polishing: (**a**) friction coefficient curve, (**b**) average friction coefficient.

Figure 12a,b depict the curves of the friction coefficient over time and the average friction coefficient of the sample surface after three distinct polishing processes, respectively. Upon examination of the figures, it becomes evident that the friction coefficients experienced rapid escalation during the initial stages of the friction experiments, with the most pronounced increase observed for the laser polished and laser-electrochemical hybrid polished samples. As the friction experiments progressed, the friction coefficient of each sample gradually stabilized. The findings reveal that the average friction coefficients of the pristine surface and the surfaces treated by electrochemical, laser, and hybrid processes amount to 0.47, 0.47, 0.45, and 0.41, respectively. Notably, the friction coefficients are directly linked to the roughness of the material surface, with higher roughness corresponding to higher friction coefficients.

#### 3.6.2. Specific Wear Rate

**Figure 13:**
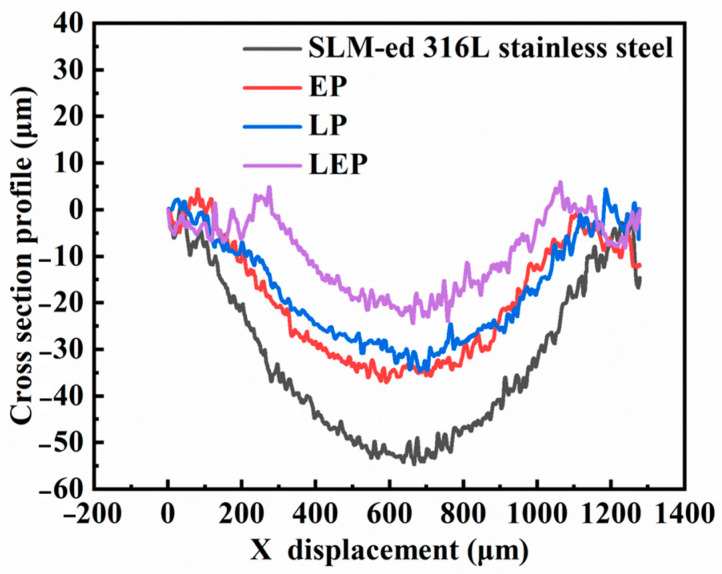
Cross-sectional outline of wear track of SLM-formed 316L SS before and after polishing.

Figure 13 displays the cross-sectional profiles of the surface wear marks on the untreated and polished samples of SLM-formed 316L SS. The results indicate that the depths of the surface wear marks on the original sample and the surfaces following electrochemical, laser, and hybrid process polishing are approximately 50 μm, 30 μm, 28 μm, and 20 μm, respectively. It is noteworthy that the wear marks on the polished specimens are all shallower than those on the initial sample surface. The laser-electrochemical hybrid polishing samples exhibited the lowest depth of wear marks, attributable to their reduced surface roughness and enhanced microhardness. In contrast, the electrochemical polishing samples demonstrated increased roughness and decreased microhardness, resulting in a greater depth of wear marks compared to the other two polishing processes.

The wear rate of the specimen is calculated as follows [35]:

(1) W=ΔV/(L×D)

where Δ*V* represents the volume of wear marks in mm^3^, *D* is the sliding distance in meters, and *L* denotes the applied load in Newtons. The wear volume Δ*V* is calculated as:

(2) $$\Delta V=\frac{Lh}{6b}(3h^{2}+4b^{2})$$

In the above equation, *L* is the length of the abrasion mark (mm), *h* is the depth of the abrasion mark (mm), and *b* is the width of the abrasion mark (mm), where *h* and *b* are both measured with a laser confocal microscope (Figure A2).

**Figure 14:**
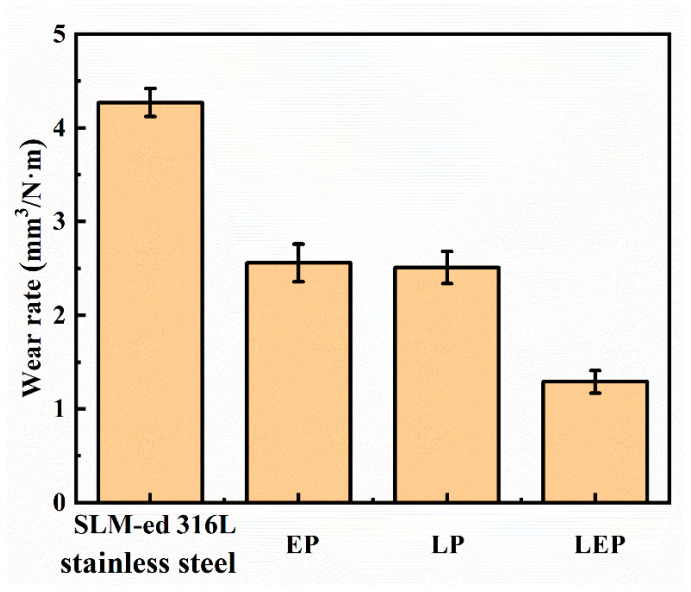
Specific wear rate of SLM-formed 316L SS before and after polishing.

As depicted in Figure 14, the wear resistance of the polished surfaces exhibited notable improvement in comparison to the original sample surface. Notably, the specimens subjected to the hybrid polishing process demonstrated the highest level of wear resistance. This enhancement can be primarily attributed to the surface roughness and microhardness improvements achieved through the polishing process.

#### 3.6.3. Wear Morphology

**Figure 15:**
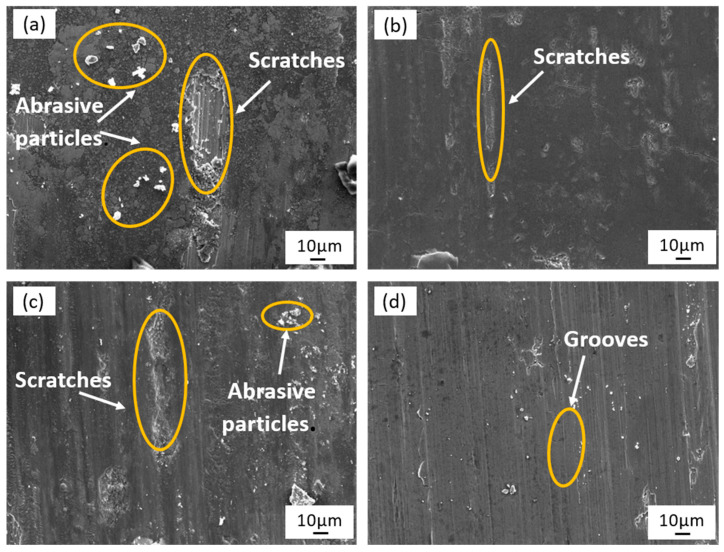
Morphology of worn surface before and after polishing: (**a**) original sample, (**b**) laser-polished sample, (**c**) electrochemically polished sample, (**d**) laser-electrochemical hybrid polished sample.

Figure 15 illustrates the surface micro-morphology of the specimen after wear. The presence of abrasive particles and furrow scratches on the original specimen surface following abrasion (Figure 15a) can be attributed to the micro-cutting action of the steel ball during the experimental process, resulting in plastic deformation and material spalling. The spalled material is then refined into small-sized abrasive particles due to the grinding action of the steel ball, and the wear mechanism at this point is mainly abrasive wear and adhesive wear. In the case of the laser-polished specimen, the size of the furrows on the surface due to abrasion is significantly reduced (Figure 15b), with a drastic reduction in the number of abrasive grains. This reduction can be attributed to the decrease in surface roughness and the increase in hardness of the material, and the abrasion mechanism at this time is mainly adhesive wear. On the other hand, the degree of abrasion on the surface of the electrochemically polished sample falls between the above two specimens. Although its roughness is comparable to that of the laser-polished sample, it is exacerbated by the lower surface hardness (Figure 15c), and the wear mechanism is mainly adhesive wear and abrasive grain wear. In contrast, the hybrid-polished sample exhibits the smallest size of abrasive marks and fewer abrasive grains on the surface, resulting in a flat and relatively smooth surface (Figure 15d), and the wear mechanism is mainly slight abrasive wear. These findings further validate that the laser-electrochemical hybrid polishing process effectively reduces surface roughness and enhances the microhardness of the material.

## 4. Conclusions

In this study, the laser-electrochemical hybrid process was employed to polish SLM-formed 316L SS, with a focus on investigating the impact of various process parameters on surface roughness and micromorphology. Additionally, a comparative analysis was conducted among the laser, electrochemical, and laser-electrochemical hybrid processes with regard to surface roughness, microstructure, and mechanical properties of the polished material. The key findings are summarized as follows:

(1) Increasing the laser power and polishing time both contribute to improved surface flatness. However, excessively high laser power can generate bubbles, resulting in severe scattering and uneven surface polishing. Similarly, prolonged polishing time may cause excessive corrosion of the material surface;
(2) The hybrid process demonstrates higher polishing efficiency and superior surface quality than individual laser and electrochemical polishing methods. Furthermore, to some extent, the surface hardness of stainless steel is enhanced through the hybrid process, leading to the lowest coefficient of friction and specific wear rate in friction and wear tests. This process also results in reduced surface abrasion and superior wear resistance;
(3) The best laser-electrochemical hybrid polishing results were obtained when the laser power was 16 W, the scanning speed was 100 mm/s, and the current was 5 A. Compared with the original samples, the roughness was reduced by 83.4%, the microhardness was increased by 4%, and the specific wear rate was reduced by 70%;
(4) The laser-electrochemical hybrid process exhibits promising potential for applications in the efficient and high-quality surface polishing of additive-manufactured metal components.

## Figures and Tables

**Table 1:** Main chemical composition of 316L SS powder.

Element	Ni	Cr	Mo	C	Mn	Si	Fe
Percent (wt%)	10.72	16.96	2.44	0.01	0.73	0.51	Bal.

**Table 2:** Processing parameters for comparison experiments.

Samples	P/W	V/(mm/s)	f/kHz	n	I/A	Time/min
LP	16	100	40	2	-	-
EP	-	-	-	-	5	3
LEP	16	100	40	2	5	3

**Figure A1:**
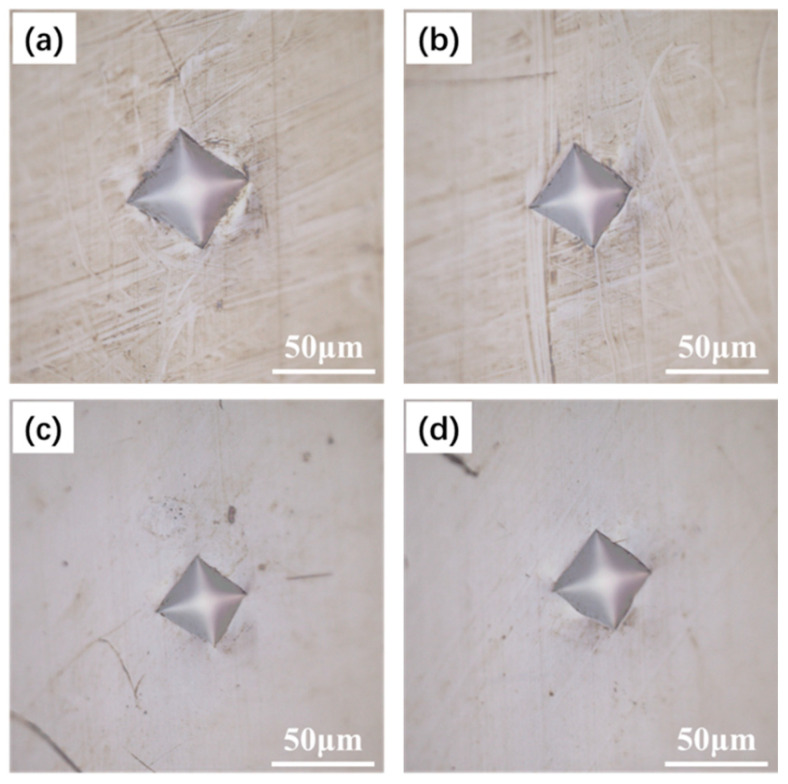
Microhardness test picture: (**a**) SLM-ed 316L stainless steel sample, (**b**) EP sample, (**c**) LP sample, and (**d**) LEP sample.

**Figure A3:**
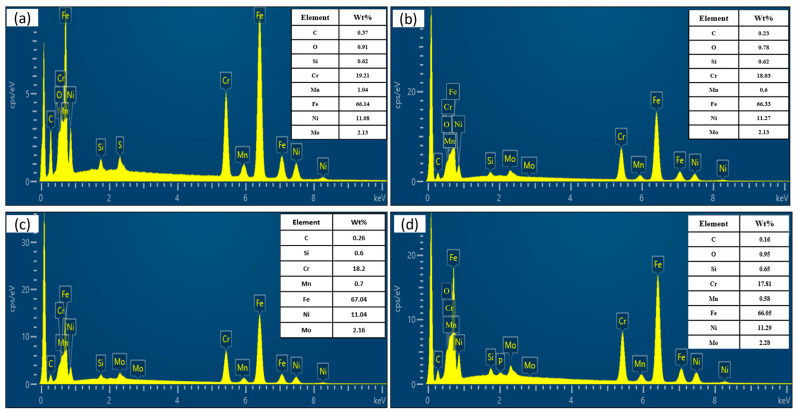
Sample EDS pictures: (**a**) original sample, (**b**) laser-polishing sample, (**c**) electrolytic-polishing sample, (**d**) laser-electrochemical hybrid polishing sample.

## Data Availability

Data are contained within the article.

## Appendix A

Figure A3 shows the EDS patterns of the sample surface for all samples, from which it can be seen that there is no obvious change in the content of the major elemental components before and after polishing. This indicates that there is no new substance produced after polishing.
